# A systematic review of existing national priorities for child health research in sub-Saharan Africa

**DOI:** 10.1186/1478-4505-3-7

**Published:** 2005-11-21

**Authors:** George H Swingler, James H Irlam, William M Macharia, Félix Tietche, Martin M Meremikwu

**Affiliations:** 1 School of Child and Adolescent Health, University of Cape Town, South Africa; 2 Directorate of Primary Health Care, University of Cape Town, South Africa; 3 Department of Paediatrics and Child Health, University of Nairobi, Kenya; 4 Department of Paediatrics, Faculty of Medicine and Biomedical Sciences, University of Yaoundé, Cameroon; 5 Department of Paediatrics, University of Calabar, Nigeria

## Abstract

**Background:**

We systematically reviewed existing national child health research priorities in Sub-Saharan Africa, and the processes used to determine them.

**Methods:**

Collaborators from a purposive sample of 20 WHO-AFRO Region countries, assisted by key informants from a range of governmental, non-governmental, research and funding organisations and universities, identified and located potentially eligible prioritisation documents. Included documents were those published between 1990 and 2002 from national or nationally accredited institutions describing national health research priorities for child health, alone or as part of a broader report in which children were a clearly identifiable group. Laboratory, clinical, public health and policy research were included. Two reviewers independently assessed eligibility for inclusion and extracted data.

**Results:**

Eight of 33 potentially eligible reports were included. Five reports focused on limited areas of child health. The remaining three included child-specific categories in reports of general research priorities, with two such child-specific categories limited to reproductive health. In a secondary analysis of Essential National Health Research reports that included children, though not necessarily as an identifiable group, the reporting of priorities varied markedly in format and numbers of priorities listed, despite a standard recommended approach. Comparison and synthesis of reported priorities was not possible.

**Conclusion:**

Few systematically developed national research priorities for child health exist in sub-Saharan Africa. Children's interests may be distorted in prioritisation processes that combine all age groups. Future development of priorities requires a common reporting framework and specific consideration of childhood priorities.

## Background

Africa experiences a huge burden of childhood disease in a context of limited resources for health care and research. Sixty five percent of the burden of disease in sub-Saharan Africa in 1990 was attributable to childhood conditions [1]. In 2001 28 of the 30 countries with the highest under-5 mortality rates were in Africa, and the under-5 mortality rate for sub-Saharan Africa was almost 25 times the average rate for industrialised countries [2]. Improvements in child health in Africa have been attributed to the findings of research – such as in vitamin A deficiency, malaria and mother to child transmission of HIV – and further research has been described as fundamental to further improvements [3]. Research is also important in guiding cost-effective policymaking. Because of severely limited resources, prioritisation of research is essential. A recent report of the status of health research in Africa highlighted the need for attention to research priority setting [4]. It cited colonial government interests as having determined priorities in the region prior to independence, and suggested that in the post-independence period priority setting has been haphazard, and determined by institutions or individuals rather than based on country or regional needs.

This study systematically reviews existing national child health research priorities in Sub-Saharan Africa, and the processes used to determine them.

## Methods

### Identification of reports on national child health research priorities

We took a purposive sample of 20 of the 45 countries in sub-Saharan Africa (corresponding to the WHO-AFRO Region, excluding Algeria). This sample was selected to provide an overall sample that, at face value, represented sub-Saharan Africa geographically, linguistically and with respect to the most important determinants of child health. It included all 15 WHO-AFRO countries in sub-Saharan Africa that had previously participated in the Africa Regional Consultative Process with respect to the status of health research in Africa (Benin, Burkina Faso, Burundi, Cameroon, Cote d'Ivoire, Ethiopia, Guinee Conakry, Kenya, Mali, Mauritius, Nigeria, Senegal, South Africa, Tanzania and Zambia) [4]. For this study, five more countries (Angola, Chad, Democratic Republic of Congo, Ghana and Zimbabwe) were added to improve on representiveness. The total population in the countries included in this survey represented 80% of the total population of sub-Saharan Africa [5], and 76% of the MEDLINE-indexed research output on child health from the region over the study period.

For the primary search, collaborators in each country identified personnel in national Ministries of Health, universities, research institutes, non-governmental organisations and funding organisations who had participated in national health research forums, or who were considered to be knowledgeable about national health research priorities by virtue of their professional positions. These 'key informants' were then surveyed by means of a pre-piloted mailed self-administered questionnaire. They were first contacted by telephone, or in person when judged appropriate. Country collaborators collected questionnaires, clarified responses in the questionnaire and followed up non-respondents (generally first by mail, fax or email, with non-respondents thereafter followed up by telephone and direct contact). They also obtained and forwarded to the authors copies of all available reports that potentially met the study inclusion criteria.

MEDLINE was also searched in order to locate additional indexed reports from the sampled countries. The MeSH terms "Health Services Research", "Health Priorities", "Health Policy", "Nutrition Policy", "Policy Making", text words "policy", "research", and "priorit*", and MeSH country terms, were used.

A secondary search was performed for Essential National Health Research (ENHR) reports on priority setting processes from all 45 countries in the sub-region, whether sampled for the primary analysis or not. This broad search was restricted to ENHR reports because the central promotion and monitoring of the process was expected to enable reliable identification of reports from central sources. The standard process recommended for setting priorities and compiling reports was also expected to enable a comparison of sampled and non-sampled countries. The search was augmented by information obtained from the website of the Council on Health Research for Development (COHRED) [6], and from members of the African Health Research Forum, COHRED, ENHR focal points, and the authors' personal networks.

### Inclusion criteria for reports on national child health research priorities

Documents eligible for inclusion were all reports or other formal documents, dated from 1990 to 2002, from a national or nationally accredited institution, describing national research priorities for child health, alone or as part of a broader report in which children were an identifiable group. There was no language restriction.

Childhood covered any age from birth to 18 years. The term "research" referred to basic (laboratory), clinical, public health or policy research. "Child health" included both health or nutritional conditions (e.g. diarrhoea, Vitamin A deficiency) or determinants of child health (e.g. breastfeeding, tobacco smoke), provided that studies of the determinants included health-related outcomes or associations. Reports could prioritise both between different health conditions, within a single health condition, or between risk factors for childhood disease or malnutrition.

### Critical assessment of reports on national child health research priorities

The quality of reports was assessed using criteria modified from those proposed by the Global Forum for Health Research for consideration when setting health research priorities [7]. viz. consideration of i) burden of disease; ii) determinants of disease; iii) the burden of determinants of disease; iv) the present level of knowledge, and v) the cost-effectiveness of interventions. In addition, the interest groups participating in the setting of priorities were recorded for use in the determination of the breadth of participation.

Potentially eligible reports identified by country collaborators were independently assessed for inclusion by two reviewers, with disagreements resolved through consensus.

### Data extraction and statistical analysis

Pre-specified data were extracted independently by two reviewers onto a pre-designed data extraction form. Disagreements were resolved by consensus. French language documents were examined by French speaking reviewers. Data extracted included date of report, the presence of the inclusion criteria, the health conditions and determinants considered, interest groups and institutions participating, funders and the presence of the quality criteria specified above. Listed priorities were extracted verbatim for later analysis, translated where necessary from French into English by a bilingual author (FT).

Quantitative synthesis was not attempted.

### Ethical approval

Ethical approval was obtained from the Research Ethics Committee of the University of Cape Town (ref 251/2002).

## Results

**Figure 1:**
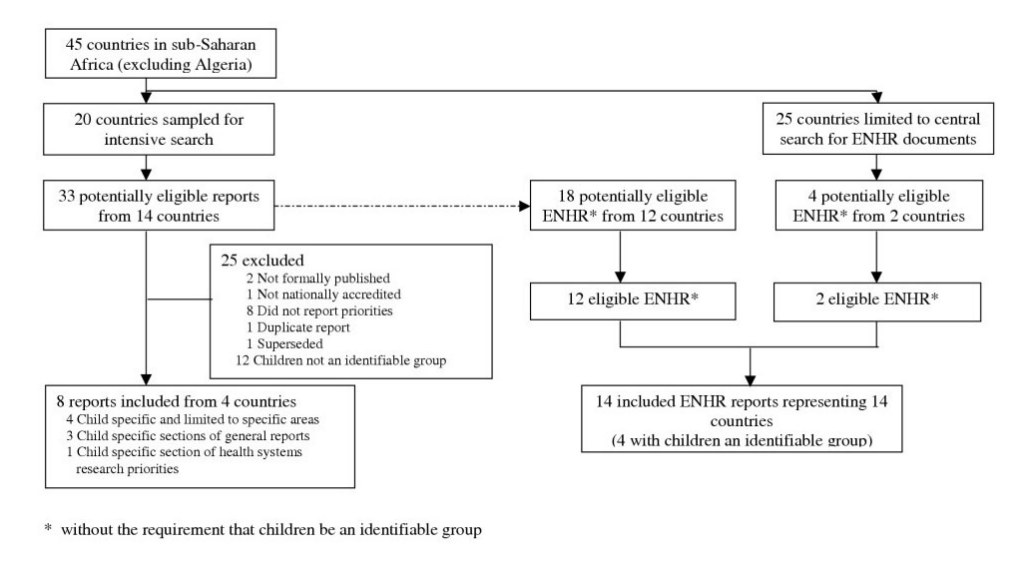
Identification of studies.

Two hundred and fifteen key informants responded to the questionnaire survey. Thirty three potentially eligible reports were identified from 14 of the 20 sampled countries (Figure 1). The 14 countries from which reports were identified generated 99.4% of the MEDLINE-indexed research output on child health from the sample for the period under review. Eight studies met the study inclusion criteria [8-15] (Table 1). Of the 25 excluded reports, 12 would have been eligible if priorities had been reported with children as an identifiable group [16-28]. Characteristics of included studies plus those that would have been eligible if children had been an identifiable group are shown in Table 2.

**Table 1:** Reports identified

	**Included**		**TOTAL**
	**Yes**	No*	
Burkina Faso		3 (3)	3
Cameroon		1 (1)	1
Cote d' Ivoire		1 (0)	1
Ethiopia		1 (0)	1
Ghana		1 (1)	1
Guinea		1 (1)	1
Kenya		2 (1)	2
Mali		1 (1)	1
Nigeria	2	1 (0)	3
Senegal	1		1
South Africa	4	10 (2)	14
Tanzania		2 (1)	2
Zambia	1		1
Zimbabwe		1 (1)	1
**TOTAL**	**8**	**25 (12)**	**33**

* The numbers in brackets represent reports that would have been eligible if child priorities had been separately identifiable

**Table 2:** Included reports

**Name of Report**	**Country**	**Date**	**Scope**
***Child health priorities separately identifiable***			
Priorities for Health Research in Nigeria.[8]	Nigeria	2001	General
Handbook for Health Systems/Operations Research at Local Government Level.[9]	Nigeria	1993	Health systems
Interim findings on the National PMTCT Pilot Sites.[10]	South Africa	2002	HIV/AIDS
Workshop on an integrated policy for school health.[11]	South Africa	1997	School health
Chronic Disease of Childhood. Workshop proceedings.[12]	South Africa	1999	Chronic diseases
Saving Babies 2001. Second perinatal care survey of South Africa.[13]	South Africa	2001	Perinatal
Programme National de Recherche en Santé (National Programme of Health Research).[14]	Senegal	2001	General
Zambia National Health Research Agenda.[15]	Zambia	1999	General
***Child health priorities not distinguished from adult priorities***			
Les programmes d'intervention nutritionnelle au Burkina Faso (Nutritional intervention programmes in Burkina Faso).[16]	Burkina Faso	2001	Nutrition
Actes du Premier Symposium sur la Recherche Nationale en Santé au Burkina Faso (Proceedings of the first symposium on national essential health research in Burkina Faso).[17]	Burkina Faso	1997	General
Plan National d'Action pour la Nutrition [version revisée]. (National Action Plan for Nutrition [revised version]).[18]	Burkina Faso	2001	Nutrition
Recommendations of the National Symposium on Medical Research in Cameroon.[19]	Cameroon	2002	General
Medium Term Health Strategy: Towards Vision 2020.[20]	Ghana	1995	General
Atelier de réactualisation des priorités nationales en matière de recherche en santé en République de Guinée (National workshop on definition of health research priorities).[21]	Guinea	2000	General
Conceptual Framework for Essential National Health Research in Kenya.[22,23]	Kenya	1994	General
First National Symposium on Health Research Priority Setting in Mali.[24]	Mali	2001	General
Foresight Health Report.[25]	South Africa	1999	General
Proceedings of the First Essential National Health Research Congress on Priority setting.[26]	South Africa	1996	General
Tanzania Essential National Health Research Priority Setting Workshop. Final Report.[27]	Tanzania	1999	General
The Essential National Health Research.[28]	Zimbabwe	1995	General

The extent to which included reports satisfied the pre-specified quality criteria is shown in Table 3, together with reports that would have been eligible if priorities had been reported with children as an identifiable group. Six (30%) of 20 reports satisfied three or more of the five quality criteria, with the number of criteria met ranging from zero to five. Table 4 lists the interest groups participating in the prioritisation processes of the reports

**Table 3:** Quality criteria met by included reports

	Included reports (%), n = 8			Reports that would have been eligible without the requirement of child-specific priorities (%), n = 20		
Consideration of:	Yes	No	Unclear	Yes	No	Unclear
burden of disease	6 (75)	2 (25)	0 (0)	14 (70)	4 (20)	2 (10)
determinants of disease	5 (62.5)	2 (25)	1 (12.5)	10 (50)	8 (40)	2 (10)
burden of determinants of disease	2 (25)	5 (62.5)	1 (12.5)	2 (10)	16 (80)	2 (10)
existing knowledge	4 (50)	4 (50)	0 (0)	10 (50)	10 (50)	0 (0)
cost-effectiveness of interventions	2 (25)	5 (62.5)	1 (12.5)	5 (25)	14 (70)	1 (5)

**Table 4:** Interest groups participating in the prioritisation processes of included reports

	Included reports (%), n = 8			Reports that would have been eligible without requirement of child-specific priorities (%), n = 20		
	Yes	No	Unclear	Yes	No	Unclear
Researchers	6 (75)	1 (12.5)	1 (12.5)	16 (80)	2 (10)	2 (10)
Institutions of learning	5 (62.5)	2 (25)	1 (12.5)	15 (75)	3 (15)	2 (10)
Health managers	6 (75)	1 (12.5)	1 (12.5)	16 (80)	2 (10)	2 (10)
Policy makers	6 (75)	1 (12.5)	1 (12.5)	17 (85)	2 (10)	1 (5)
Non-governmental organisations	3 (37.5)	4 (50)	1 (12.5)	12 (60)	6 (30)	2 (10)
Consumers	2 (25)	5 (62.5)	1 (12.5)	9 (45)	9 (45)	2 (10)

Four of the eight studies with children as an identifiable group addressed limited areas of child health only i.e. mother to child transmission of HIV, school health, chronic diseases of childhood, and perinatal care [10-13]. All four were from South Africa. The remaining four reports, from Nigeria (two), Senegal and Zambia, covered research priorities for both children and adults and included specific categories of child health [8,9,14,15]. One of these four reports included a section dealing with general child health [15], one dealt with maternal and child health in a report limited to health systems research priorities [9], one with "Adolescent health and sexuality" [8], and one with "Pregnancy and delivery, pregnancy and oral cavity diseases, mother and child care, adolescent health" limited entirely to the pre-natal period [14]. The wide variation in the areas of interest of the eight reports in the primary analysis precluded any attempt at synthesis of priorities.

Eighteen reports from 12 of 20 sampled countries reported ENHR processes (Figure 1). Of the other eight countries in the sample, there was no record of ENHR processes having been initiated in three countries, and confirmation of no report of priorities in a fourth [6]. ENHR documents were thus identified from 12 (75%) of the 16 countries from which they were potentially available by the primary search. The secondary search for ENHR reports from all 45 countries in the sub-region identified four potentially eligible reports from two countries not included in the primary sample (Malawi and Uganda), two of which were found to be eligible. In total therefore, 14 reports from 14 countries met the secondary inclusion criteria; two from non-sampled countries (Malawi and Uganda) [8,14,15,17,19-24,26-30]. All of the reports dealt with general priorities. Four reports covering research priorities for both children and adults included specific categories on child health (from Nigeria, Senegal, Uganda and Zambia) [8,14,15,30]. Only two reports listed research priorities for child health as a whole. Except for possible overlap in the area of nutritional interventions, there were no specific priorities common to both [15,30]. The two other reports were those described above that dealt with reproductive health [8,14].

Despite a standard ENHR process for prioritisation, there was wide variation in the frameworks for the categorisation of research priorities, and in the number of categories and sub-categories in each report. Some reports listed only specific health conditions, while others categorised by other, but varying, frameworks such as health systems, public health and socio-cultural issues (Table 5). The listed priorities included one to five tiers of categorisation (median two). The number of first-tier categories of priorities varied from three to 26 (median five). Of the 10 reports with second-tier categories, the total number of priorities listed in the second-tier categories varied from 10 to 90 (median 28). Three of the 14 reports offered a scoring or ranking of research priorities. There were too few ENHR reports from non-sampled countries identified in the secondary search to compare priorities of sampled with non-sampled countries.

**Table 5:** Types of health-related categories listed in first- and second-tier headings of reports

	First tier headings (n = 14)	Second tier headings (n = 10)
Specific health conditions	11 (78.6%)	10 (100%)
Determinants of health conditions	0 (0%)	5 (50%)
Nutritional issues	6 (42.9%)	9 (90%)
Public health issues	12 (85.7%)	10 (100%)
Research issues	3 (21.4%)	3 (30%)

## Discussion

This study aimed to provide as valid and replicable an overview of existing child health research priorities as possible, and to describe the processes whereby the priorities were set. To do so, a pre-specified systematic approach was followed, adapted from the process now widely accepted in healthcare practice [31,32]. This approach to health research priorities does not adequately address nuances of meaning and local context, and thus represents an incomplete picture. Although insufficient in itself, we suggest that this analytical approach is an essential component of the assessment of research prioritisation processes, particularly because of the shortcomings of existing processes identified by this study.

Prioritisation reports were identified from the 14 countries that generated 99.4% of the MEDLINE-indexed child health research output of the entire sample of 20 countries. If it is assumed that the countries producing the bulk of published research also generate the bulk of prioritisation processes, the study appears to have identified prioritisation reports from the countries in which they might be expected to exist. ENHR documents were obtained from 12 of 16 countries from which they were potentially available, representing a minimum 75% success rate in identifying ENHR documents. The sample itself was limited to 20 of the 45 countries in the WHO-AFRO region (excluding Algeria), representing 80% of the total population under study and 76% of the MEDLINE-indexed research articles on child health from the region. The data collected therefore appear to be reasonably representative of the population under study.

The quality of reports was variable, meeting between zero and five of the five quality criteria modified from the approach suggested by the Global Forum for Health Research for the development of priorities [7]. However, these criteria were not proposed primarily as quality assessment criteria and have not to our knowledge been validated as such. The representation on prioritisation teams was generally broad.

The most striking finding of this review is the dearth of systematically developed national research priorities in child health and child nutrition in sub-Saharan Africa. Only eight documents that offered child-specific priorities were identified from the sampled countries. In the bulk of identified prioritisation processes children were included but research priorities were not considered, or reported, in a manner that enabled a separate assessment of childhood priorities. This is concerning, given the differences in the health issues confronting children and adults, and the 65% of burden of disease in sub-Saharan Africa in 1990 attributable to conditions occurring in children [1].

Of the general reports listing research priorities for all ages, only three included child-specific categories. Two of these covered reproductive health only; one antenatal care only (i.e. pregnant children). It is unlikely that the reproductive health of children is the only priority for child health research. The prominence of reproductive health as a priority for children is probably a distortion due to overlap with adult research priorities, and suggests that children's interests are not adequately represented in processes that combine all ages. Only one (ENHR) report from the sample presented priorities for overall child health, with one additional ENHR report identified from a non-sampled country. No specific listed research priorities were common to both countries (Zambia and Uganda), possibly reflecting the variation in reporting even when a relatively standard process is used.

The secondary analysis of ENHR reports, regardless of whether children were dealt with separately, identified other obstacles to the comparison and synthesis of childhood research priorities. Even for ENHR processes, which use a standard recommended approach, the reporting of priorities varied markedly in format, with different methods of categorisation and numbers of listed priorities. The large number of research priorities listed themselves required prioritisation, but only three of 14 reports provided a ranking or weighting. These factors made comparison and synthesis of reported research priorities very difficult. A common conceptual framework for the reporting of priorities would greatly facilitate a meaningful overview of research priorities. If such a framework makes specific provision for child health and child nutrition, it could improve specific consideration and reporting of such priorities.

The shortcomings in current prioritisation processes and the difficulties encountered in synthesising research priorities raise the question of whether it is appropriate to attempt to develop sub-regional and regional research priorities by synthesising existing national priorities. This approach has the advantage of utilising existing work that is in any case necessary at national level, and of building on locally developed priorities. However, for this to be a viable approach, considerable structural changes in current processes are necessary. Challenges for the coherent development of research priorities appear to include the development of national health research system assessments that use a common conceptual framework and include specific consideration of research priorities for children.

Alternatives to this approach include regional or sub-regional application of centrally developed global research priorities, or a more qualitative and consultative synthesis of national priorities. The former involves a top-down approach that may compromise local applicability, while both processes are vulnerable to the problems of incomplete representation.

## Conclusion

Few systematically developed national research priorities for child health exist in sub-Saharan Africa. Children's interests may be distorted in prioritisation processes that combine all age groups. Future development of priorities requires a common reporting framework and specific consideration of childhood priorities.

## Competing interests

The author(s) declare that they have no competing interests.

## Authors' contributions

All authors participated in the conception and design of the study, and the acquisition, analysis and interpretation of data. GS wrote the first draft of the manuscript, and all authors participated in its critical revision for important intellectual content. All authors have seen and approved the final version. GS and JI obtained funding and co-ordinated the study. GS is the guarantor.

## Funding

The study was commissioned by the Child Health and Nutrition Research Initiative (CHNRI) of the Global Forum for Health Research and supported by a contribution from the World Bank through its grant facility to the Global Forum. CHNRI commented on the study design, but played no role in the collection, analysis or interpretation of data, the writing of the report or the decision to submit the paper.
